# Image analysis and polyphenol profiling unveil red-flesh apple phenotype complexity

**DOI:** 10.1186/s13007-024-01196-1

**Published:** 2024-05-16

**Authors:** Pierre Bouillon, Anne-Laure Fanciullino, Etienne Belin, Dimitri Bréard, Séverine Boisard, Béatrice Bonnet, Sylvain Hanteville, Frédéric Bernard, Jean-Marc Celton

**Affiliations:** 1 Univ Angers, Institut Agro, INRAE, IRHS, SFR QUASAV, F-49000 Angers, France; 2 IFO, 49140 Seiches sur le Loir, France; 3 SONAS, SFR QUASAVUniv Angers, SONAS, SFR QUASAV, Univ Angers, F-49000 Angers, France

**Keywords:** *Malus domestica*, Phenolic compounds, Image analysis, Red-flesh

## Abstract

**Background:**

The genetic basis of colour development in red-flesh apples (*Malus domestica* Borkh) has been widely characterised; however, current models do not explain the observed variations in red pigmentation intensity and distribution. Available methods to evaluate the red-flesh trait rely on the estimation of an average overall colour using a discrete class notation index. However, colour variations among red-flesh cultivars are continuous while development of red colour is non-homogeneous and genotype-dependent. A robust estimation of red-flesh colour intensity and distribution is essential to fully capture the diversity among genotypes and provide a basis to enable identification of loci influencing the red-flesh trait.

**Results:**

In this study, we developed a multivariable approach to evaluate the red-flesh trait in apple. This method was implemented to study the phenotypic diversity in a segregating hybrid F1 family (91 genotypes). We developed a Python pipeline based on image and colour analysis to quantitatively dissect the red-flesh pigmentation from RGB (Red Green Blue) images and compared the efficiency of RGB and CIEL*a*b* colour spaces in discriminating genotypes previously classified with a visual notation. Chemical destructive methods, including targeted-metabolite analysis using ultra-high performance liquid chromatography with ultraviolet detection (UPLC-UV), were performed to quantify major phenolic compounds in fruits’ flesh, as well as pH and water contents. Multivariate analyses were performed to study covariations of biochemical factors in relation to colour expression in CIEL*a*b* colour space. Our results indicate that anthocyanin, flavonol and flavanol concentrations, as well as pH, are closely related to flesh pigmentation in apple.

**Conclustion:**

Extraction of colour descriptors combined to chemical analyses helped in discriminating genotypes in relation to their flesh colour. These results suggest that the red-flesh trait in apple is a complex trait associated with several biochemical factors.

**Supplementary Information:**

The online version contains supplementary material available at 10.1186/s13007-024-01196-1.

## Introduction

Studying nature’s palette had always fascinated scientists. Mendel’s seminal laws of inheritence resulted from his studies on white and pink pea flowers. This colouration is attributed to anthocyanin, a class of phenolic compounds, that are major determinants of plant organ colours (e.g. leaves, flowers, fruits skin and flesh) with a wide range of hue variation, from orange-red to violet-blue [1]. They mostly serve to attract pollinators or seed dispersers [2] and protect against various stresses [3, 4]. In recent years, our knowledge of the mechanisms leading to the synthesis and stability of anthocyanin pigments has been enhanced by progress in genetic and biochemistry [5, 6]. The transcriptional control of the anthocyanin biosynthetic pathway has been characterized [7], highlighting a common regulatory network in Eudicots [8]. More recently, molecular engineering has enabled production of anthocyanins in non-anthocyanin plants [9]. Studies have also identified the involvement of epigenetic mechanisms in anthocyanin biosynthesis and degradation [10, 11]. Some fruits naturally exhibit red pigmentation [12] and are therefore preferential models to study red colour biochemical and phenotypical expression. The major phenolic compounds in apple are hydroxycinnamic acids, flavanols, flavonols, dihydrochalcones and anthocyanins [13].

Anthocyanins are main determinants of red colour in apple [12]. Some cultivars display an ectopic accumulation of anthocyanins in the fleshy part of the fruits leading to the ’red-flesh’ trait. This appealing phenotype [14] originates from a wild species of *Malus sieversii* [15] and is studied for its innovative aspects and potential health benefits [16, 17]. The genetic basis of flesh colour development has been characterised [18–20] in apple but current models do not explain the observed variations in intensity and pigment distribution. Studies have revealed a large diversity of phenolic compounds among red-flesh apple cultivars [21, 22], as well as their seasonal variability [23]. Environmental factors linked to anthocyanin accumulation, stability and colour expression have also been listed [16]. Among them, light intensity, water deficit and low temperature may promote anthocyanin synthesis in red-flesh cultivars. Other factors associated with expression of red colour may also be involved [24] such as pH [16, 25], interaction with other phenolic compounds leading to copigmentation events [26], or temperature during fruit storage [27].

Many plant studies rely on colour measurement [10, 28–30] and image-based phenotyping [31–34] to dissect the genetic determinism of colour development. Image-based colour measurement uses mostly colour conversion from RGB (Red Green Blue) images to different colour spaces to quantify colour variations among individuals [35]. Indeed, the RGB colour space is an additive colour model and is not suitable for colour comparison given that each colour is represented by a mix of various proportions of three distinct stimuli colours of light [36] thus, by definition, each colour channel can not be interpreted individually. Moreover, the RGB model is not a perceptually uniform space, the differences among colours in RGB space do not correspond to colour differences as perceived by humans [37]. RGB and CIEL*a*b* values have been directly used to detect quantitative trait loci (QTL) associated with colour variations in grape [38] or salvia [39]. Li et al. [40] used Gaussian estimation of pixel distributions to study colour patterning in the foliar ornamental coleus. Deep learning techniques have also been used to overcome limitations of pixel colour information (confounding effects and continuity of colours) in the case of complex plant vegetation segmentation [41]. Colour measurement can also deliver useful information on fruit intrinsic physiological changes (i.e. organoleptic, nutritional, visual or non-visual defects) [42] during fruit development, ripening and post-harvest conservation [36, 37]. For example, a convolutional neural network has been trained to evaluate fruit maturity based on starch index in apple and pear [43].

In apple, computer vision systems have been developed to sort fruits according to skin colour [44–46] or to assess the maturity stage of fruits based on skin colour measurements [47, 48]. Other systems have been designed to detect visual defects on fruits [49, 50]. An index based on colour measurements in CIEL*a*b* has been established to characterize enzymatic browning of apple slices [51, 52]. The red-flesh trait in apple is difficult to assess visually due to non-homogeneous and non-continuous colour distribution leading to pigmentation pattern and variations in red hue. Various procedures to evaluate red-flesh colour based on imaging techniques have already been developed in apple [53, 54]. These methods include the evaluation of anthocyanin contents derived from images of red-flesh apple fruit section [55], the estimation of average overall red colour [54] and the non-destructive analysis of fruits to predict red-flesh colour using interactance spectroscopy [56, 57]. However, current methods based on spectroscopy are less predictive than destructive methods [57], while most RGB image-based methods do not consider evaluation of non-homogeneous surface [38]. Moreover absolute quantification of anthocyanins is laboratory-based and involves costly and time-consuming steps.

In this study, we developed a new method to dissect flesh colour from fruit section images acquired with a RGB scanner and the conversion of RGB images into CIEL*a*b* colour space. This method was applied to identify biochemical factors involved in colour variations in a F1 apple progeny segregating for the red-flesh trait and displaying a large variability in red-flesh phenotypes. Colorimetric variables from the CIEL*a*b* colour space were used to build a Partial Least Squares regression model allowing the identification of the main biochemical factors controlling pigmentation variations in red-flesh apple.

## Methods

### Experimental approach

Our objective was to develop a robust method to differentiate apple fruits based on colour parameters using image analysis. Colour descriptors from RGB and CIEL*a*b* colour space were used to discriminate genotypes from a F1 apple progeny segregating for the red-flesh trait. Major phenolic compounds potentially involved in red pigmentation were quantified, and correlations among these compounds and the colour descriptors were estimated. Finally, models based on colours descriptors (a*, b* and hue) variations were established to identify biochemical factors involved in red-flesh pigmentation.

### Plant material

Fruit harvest was conducted from late August to mid-October in 2022 and 2023 in IFO orchard (L’Anguicherie, 49,140 Seiches-sur-le-Loir, France/ GCS: 47°37’52.5”N 0°19’38.4”W). Our study was carried out on 91 genotypes from a F1 hybrid family segregating for the red-flesh trait (plantation year: 2017). Each genotype is represented by one tree grafted on M9 Pajam®2 Cepiland C.O.V. For each genotype harvested at maturity (brix values varying from 13 to 22; starch index between 6 and 8), four representative fruits were dedicated to image analysis for estimation of the red-flesh intensity and distribution, while four other fruits were sampled for the quantitation of phenolic compounds. Fruits that were positioned in the middle of each tree, with similar exposure to light, same developmental stage, and similar diameter were harvested preferentially to limit intra- and inter-tree bias. Image acquisition was performed for fruits harvested in 2022 and 2023 while phenolic compounds quantification was carried out only on fruits harvested in 2022. Colour descriptors and biochemical factors were estimated for four apples per genotype.

### Image analysis

#### Evaluation of fruit flesh colour

Prior to image acquisition, fruits were visually classified into six intensity classes according to a red colour scale (from white flesh 0 to dark red-flesh 5, Figure S1). The mean of the four fruits provided the colour index of each genotype and enabled image analysis conformity evaluation.

#### Image acquisition

**Fig. 1:**
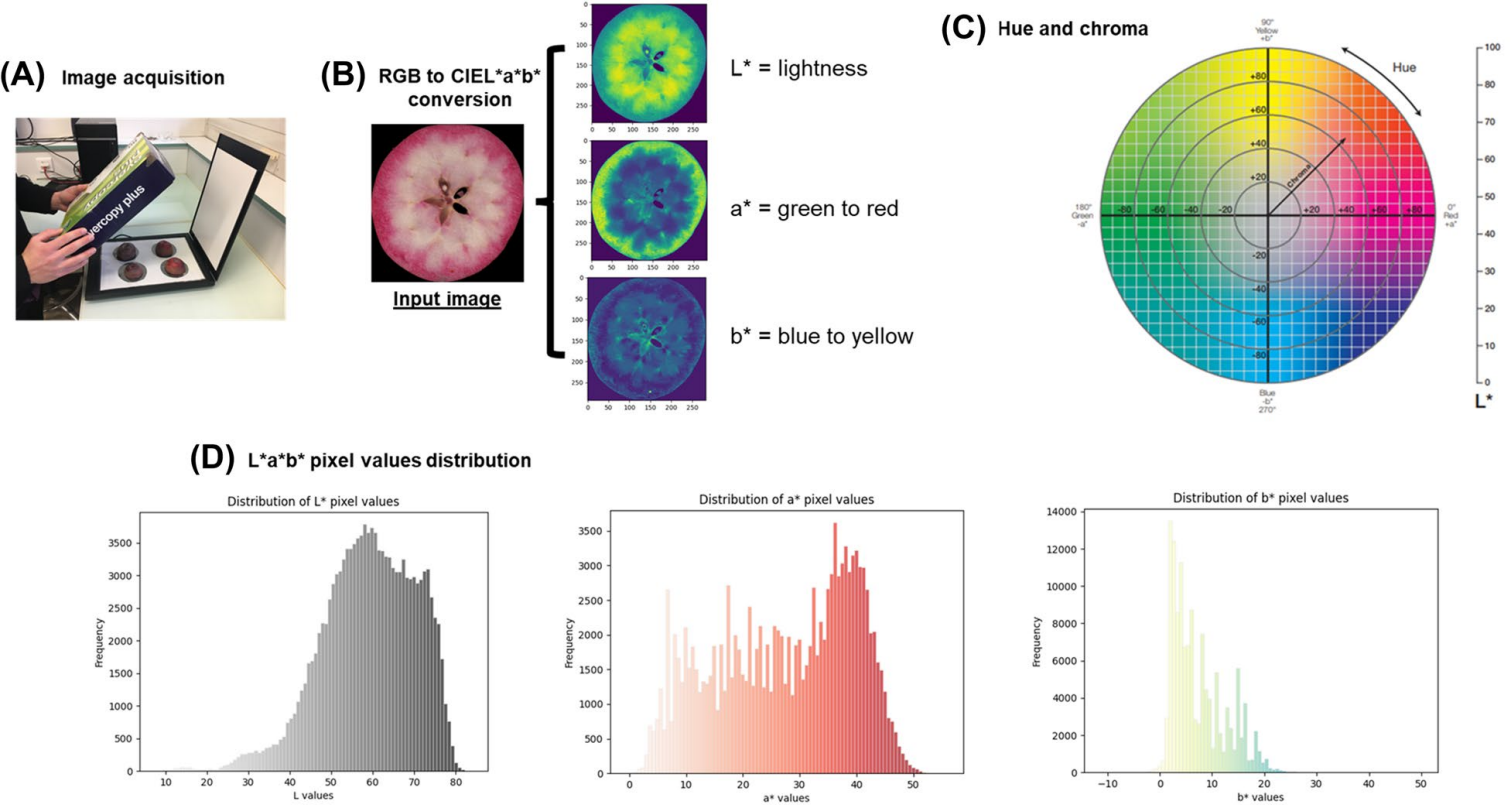
Overview of the analysis pipeline **A**: image acquisition is performed with a RGB flatbed scanner. **A** shade box ensures reproducible lightning conditions. **B**: RGB images of apple sections are converted to CIEL*a*b* colour space. This conversion is nonlinear. Therefore, the RGB colour space was transformed into CIEXYZ before CIEL*a*b* conversion. **C**: chroma and hue are estimated from a* and b* colour descriptors. **D**: statistical descriptors are calculated (mean and standard deviation) on CIEL*a*b* pixel distribution to approximate colour for a non-homogeneous surface

In an affordable phenotyping approach [58, 59], images of fruits were acquired using a RGB flatbed scanner Canon LIDE 400 (Fig. 1A). A transversal section of each fruit was scanned immediately after cutting to avoid enzymatic browning. A shade box was positioned upon the system to reduce external light pollution. Image acquisition was achieved with the IJ scan utility software and images were stored in png format.

#### Image processing

An in-house object detection pipeline was used to obtain individual apple section. Firstly, an Otsu’s thresholding was performed to separate apple objects from the background. Then, a Connected Component Analysis isolated each apple object and drew a bounding box around the object contours, giving four apple section images from a single file. Final images were converted in.png format and stored for further analysis. Apple section images resolution are approximately 400*400 pixels.

#### Image analysis

The image analysis pipeline was written in Python. Firstly, RGB images of apple sections were converted to CIEXYZ, then to CIEL*a*b* with default parameters Illuminant = “D65” (Fig. 1B) using Scikit-image [60]. The image acquisition system provided repeatable lightning conditions and ensured relative comparison between genotypes. CIEL*a*b* is a device-independent and uniform colour space derived from CIE XYZ space. In the CIEL*a*b* colour space, L* is associated with the lightness of the colour (L* = 0 means black and L* = 100 refers to white). a* is related to the colour tonalities from red (+) to green (−), and b* is associated with yellow (+) to blue (−) colour variations [61]. Basic statistical descriptors of pixel values distribution were calculated (mean and standard deviation) with Numpy library [62] (Fig. 1C). Hue angle was calculated with the formula h = arctan$(\frac{a*}{b*})$ and chroma $\hbox {C}* = \sqrt{a^2+b^2}$ (Fig. 1D).

### Determination of phenolic content

#### Metabolites extraction

Fruits without peel were cut and two flesh pieces from the sun-exposed side of the fruit were selected (about 5 gs fresh weight per fruit). Samples were immediately frozen in liquid nitrogen, then stored at − 80°C and freeze-dried. This dried material was crushed into a fine and homogeneous powder and stored until analysis. About 50 mg of this powder was precisely weighed in a 2 mL microtube. Polyphenols extraction was adapted from [63]. Samples were extracted during 30 min in an ultrasonic bath with 1.5 mL of methanol (MeOH) containing 5% acetic acid (v/v) and spiked with 2 $\upmu$g of internal standard (IS) in order to control the extraction recovery. After centrifugation (14,800 rpm/10 min), supernatants were filtered with RC filters (0.2 $\upmu$m/13 mm; Macherey-Nagel) and transferred to LC vials.

#### Phenolic compound detection and quantification

First, pools of ten hybrids from the F1 progeny were analyzed using an ACQUITY quantitative ultra-high performance liquid chromatography (UPLC) H-Class Series system coupled to a Xevo G2-XS mass spectrometer (Waters) in order to identify the metabolites by comparing the retention time and *m/z* data from literature and/or with data obtained from authentic standards. The UPLC-Q-TOF was equiped with a quaternary solvent manager, a sample manager and a column compartment as chromatographic part and coupled to a quadrupole time-of-flight (Q-TOF) mass spectrometer. The data was processed with the Progenisis QI software (Waters, Elstree, UK). Ten phenolic compounds were identified with level 1 of confidence according to [64] and appeared to be the major phenolic compounds in our samples. These ten phenolic compounds were quantified in all our genotypes. Water content was calculated based on fresh weight (FW) and dry weight (DW) with the following equation: WC = 1-$\frac{DW}{FW}$. pH was measured using a pH-meter Seven Compact S210 (Metler Toledo) after fruit powder re-suspension to match corresponding individual fruit water content.

The calibration curves were set to the concentration range expected for each compound and their linearity was assessed by injecting 6 levels of calibration standards in three replicates. Residuals (difference between nominal concentration and calculated concentration by the linear model) and their distribution (normally distributed around the mean) were monitored (Minitab 19 software). Precision was evaluated by repeated analysis of standards within different analytical batches. Limit of detection (LOD) and limit of quantification (LOQ) were defined as 3- and 10- fold the signal-to-noise ratio, respectively. When considering samples with area under the curve (A.U.C) inferior to LOD, they were expressed as LOD/2 to limit bias for statistical analysis.

Each sample was spiked with the same level of IS at the start of the extraction procedure in order to evaluate the recovery of extraction. The precision of the extraction and quantification process was evaluated from the analysis of 5 replicates of the 2 parents of the segregating F1 progeny.

**Table 1:** Method parameters

Compound	Retention time (min)^a^	UV detection $\lambda$ max (nm)	LOD (ng)^b^	LOQ (ng)^c^	Linearity range (ng)^d^	R^2^
Procyanidin B1	12.75 ± 0.12	279	0.24	0.47	0.9 – 47.3	1.000
(+)− catechin	13.45 ± 0.34	279	0.50	1	2.5 – 99.8	0.998
Procyanidin B2	16.26 ± 0.07	279	0.24	0.48	9.6 – 478.5	1.000
(-)− epicatechin	16.74 ± 0.25	279	0.25	0.49	4.9 – 247.3	0.996
Chlorogenic acid	17.44 ± 0.39	325	0.49	0.99	98.9 – 1483.5	0.997
Cyanidin 3-galactoside	18.62 ±0.11	512	2.40	4.7	4.7 – 236.3	0.999
4-*p*-coumaroylquinic acid	20.79 ±0.34	312	0.13	0.25	5 – 250	1.000
Procyanidin C1	21.99 ±0.33	279	0.90	2.3	4.6 – 232	1.000
Phlorizin	33.41 ±0.11	284	0.09	0.23	0.9 – 46.2	0.998
Quercetin 3-galactoside	36.21 ±0.17	350	0.24	0.47	0.5 – 9.5	0.976
Daidzein (IS)	38.73 ± 0.19	250	0.10	0.25	2.5 – 49	0.997

^a^Values are mean ± SD (n=3)

^b^The signal-to-noise was set to 3:1

^c^The signal-to-noise was set to 10:1

^d^Suitable for samples

#### UPLC-UV analysis

Methanol (HiPerSolv CHROMANORM for LC-MS) was purchased from VWR. Acetic acid, formic acid and dimethylsulfoxide (analytical reagent grade) were purchased from Fisher Scientific. Ultrapure water was obtained from a MilliQ advantage A10 purification system (Millipore). Quercetin 3-galactoside, cyanidin 3-galactoside chloride, procyanidin B1, procyanidin B2, procyanidin C1, (+)-catechin, (−)-epicatechin, chlorogenic acid and phlorizin standards were purchased from Extrasynthese. 4-*p*-coumaroylquinic acid was purchased from Ambinter. Daidzein (internal standard) was purchased from Molekula. All standards were furnished with a certificate of analysis. Stock solutions of procyanidin B1, procyanidin B2, procyanidin C1, phlorizin, quercetin 3-galactoside and daidzein were prepared in DMSO at a concentration of 5 mg.mL^-1^. Stock solutions of (+)-catechin and (−)-epicatechin were prepared in MeOH at a concentration of 5 mg.mL^-1^. Stock solutions of chlorogenic acid and 4-*p*-coumaroylquinic acid were prepared in H_2_O at a concentration of 5 mg.mL^-1^. Stock solution of cyanidin 3-galactoside chloride was prepared in MeOH containing 5% v/v acetic acid at a concentration of 5 mg.mL^-1^. All the dilutions were then carried out in MeOH except for cyanidin 3-galactoside chloride in MeOH containing 5% v/v acetic acid. Each calibration curve was prepared using six different standards concentrations. Three replicates were used for each calibration level to determine LOD and LOQ (Table 1). Quantitative ultra-high performance liquid chromatography with ultraviolet detection (UPLC-UV) analysis was performed on a ThermoFisher Scientific Vanquish Flex UPLC system equipped with a quaternary solvent manager, a sample manager and a column compartment as chromatographic part and coupled to a variable wavelength detector as detection part. A sample volume of 10 $\upmu$L was injected onto a ZORBAX RRHD StableBond Aq column (2.1 x 150 mm; 80 Å; 1.7 $\upmu$m). The samples were kept at 10$\circ$C and the column was maintained at 30$\circ$C with a flow rate of 0.3 mL.min^-1^. The mobile phase consisted of 0.1% formic acid in both H_2_O (A) and MeOH (B) used in gradient mode as follows: from 5 to 15% B in 5 min, then 15 to 35 % B from 5 to 30 min, then 35 to 50% B from 30 to 35 min, then 50 to 100% B from 35 to 37 min, hold at 100% B from 37 to 39 min, and afterward the column was re-equilibrated at initial conditions during 8 min. The UV detection was measured at different wavelengths optimized for individual compounds and listed in Table 1. Data were processed using Xcalibur software.

### Statistical analysis

Broad-sense heritability (h^2^) of each colour descriptors was estimated by intra-class correlation analysis [65] with the following formula: h^2^ = $\sigma ^2_B$/($\sigma ^2_B$+$\sigma ^2_\epsilon$) where $\sigma ^2_B$ and $\sigma ^2_\epsilon$ were the individual genetic and residual variances respectively (Table 2). Genetic and individual variances were estimated from the linear model: $y_jk = \mu + B_j + \epsilon _{jk}$, with $\upmu$ the population mean of the trait (treated as a fixed effect), $B_j$ the “true effect” of the *j*th individual, and $\epsilon _{jk}$ the special environmental error. $B_j$ is assumed to be a random variable sampled from a normal distribution with a mean zero and variance $\sigma ^2_B$.

Principal Component Analyses (PCA) were carried out to investigate portability from RGB to CIEL*a*b* colour space, adequation with visual notation and ability to discriminate genotypes. Data were normalized by subtracting means and then dividing every measure by the standard deviation. Squared cosine were calculated to confirm interpretrability of explanatory variables on first components. The squared cosine shows the importance of a component for a given observation and corresponds to the square of the cosine of the angle from the right triangle made with the origin, the observation, and its projection on the component.

To further explore the relationship between the various biochemical factors and our descriptors, we built Partial Least Square (PLS) regression models (Table S4). An advantage of PLS-regression is its ability to deal with multiple colinearity compared to classical regression model [66]. The objective of PLS regression was to quantitatively dissect covariation of CIEL*a*b* colour parameters as response variable: a* / b* / hue with biochemical predictors and to identify relevant biochemical factors involved in colour expression. First, relations between colour descriptors and each polyphenol content were represented in order to identify skewed distribution that require transformation (Figure S2). We performed log-transformation for anthocyanin contents. Variable of Importance (VIP) values were then calculated following [67] with the *getVIPVn* function to extract important biochemical factors in colour expression. A variable with a VIP $< 0.8$ is a variable that appears to have a negligible impact on the model. The number of PLS components were determined as follow: a new component h is added to the model if the percentage of Y dispersion explained by component *h* is more than 1 percent. Model validation was achieved with internal validation based on calculation of Root Mean Square Error (RMSE) on test set (20% of datatest). R4.0.3 software was used to perform statistical analysis [68]. *PCA* function implements in factomineR package [69] and *ropls* package [70] were used for respectively PCA and PLS approaches. A graphical display of a correlation matrix using ggplot2 of *ggcorrplot* package [71] was chosen to represent the correlation matrix.

**Fig. 2:**
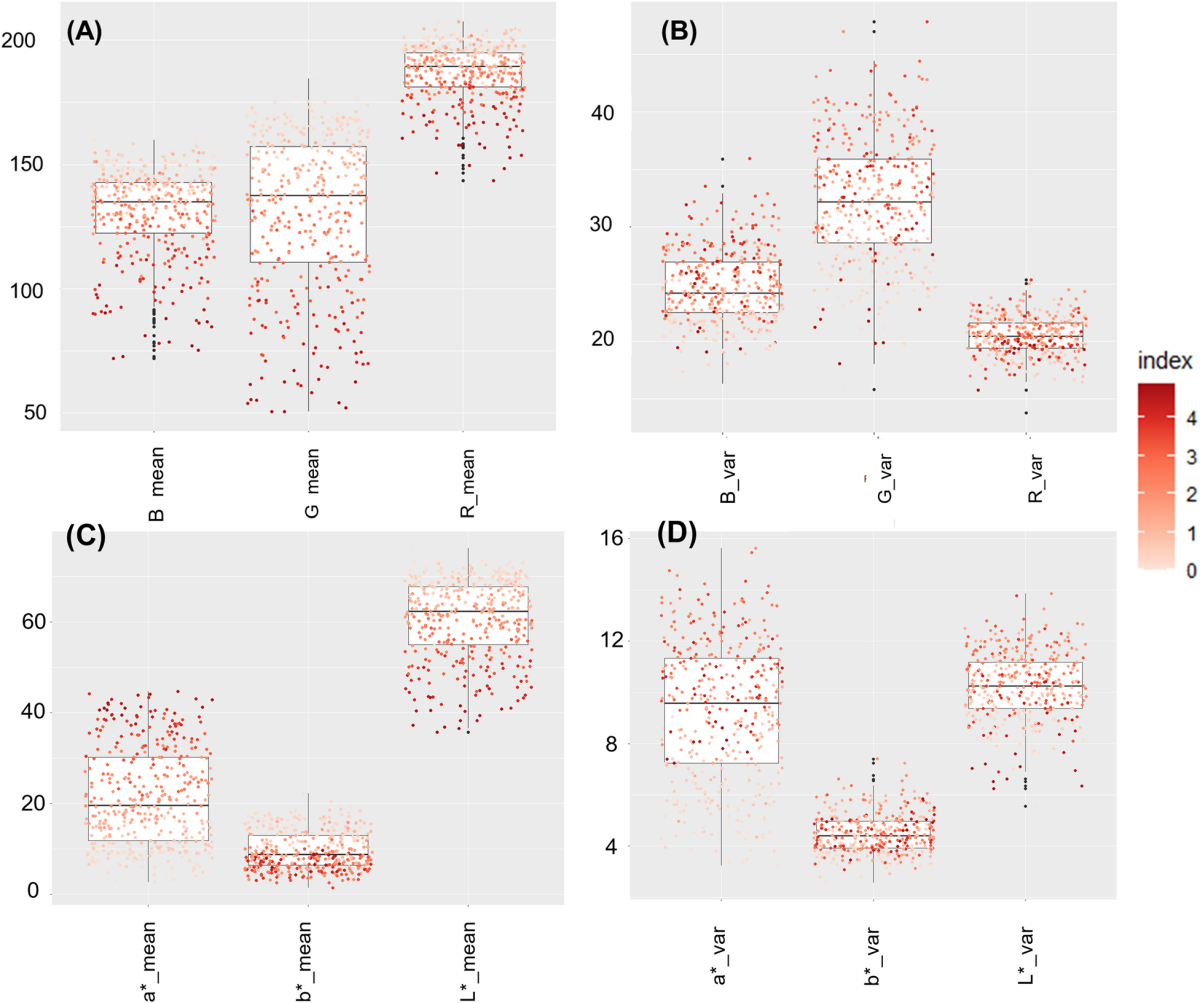
Boxplot showing colour descriptors distribution for RGB and CIEL*a*b* colour space. Colour descriptors were averaged for four apples per genotype. Dots are coloured according to the colour index. The median (denoted by a horizontal bar in the box), the 25th percentile (denoted by the bottom edge of the box), the 75th percentile (denoted by the top edge of the box) and the dots indicate single observations. **A**: Mean of RGB colour descriptors. **B**: Standard deviation of RGB colour descriptors. **C**: Mean of CIEL*a*b* colour descriptors. **D**: Standard dievation of CIEL*a*b* colour descriptors

**Table 2:** Broad-sense heritability of colour descriptors measured for fruits harvested in 2022 and 2023

Descriptor	Broad-sense heritability (h^2^)
R mean	0.755
G mean	0.783
B mean	0.763
R var	0.372
G var	0.604
B var	0.612
L* mean	0.786
a*mean	0.787
b* mean	0.744
L* var	0.59
a* var	0.649
b* var	0.392
Chroma	0.758
Hue	0.79
Index (visual notation)	0.67

## Results

### Study of colour descriptors from RGB and CIEL*a*b* spaces

**Fig. 3:**
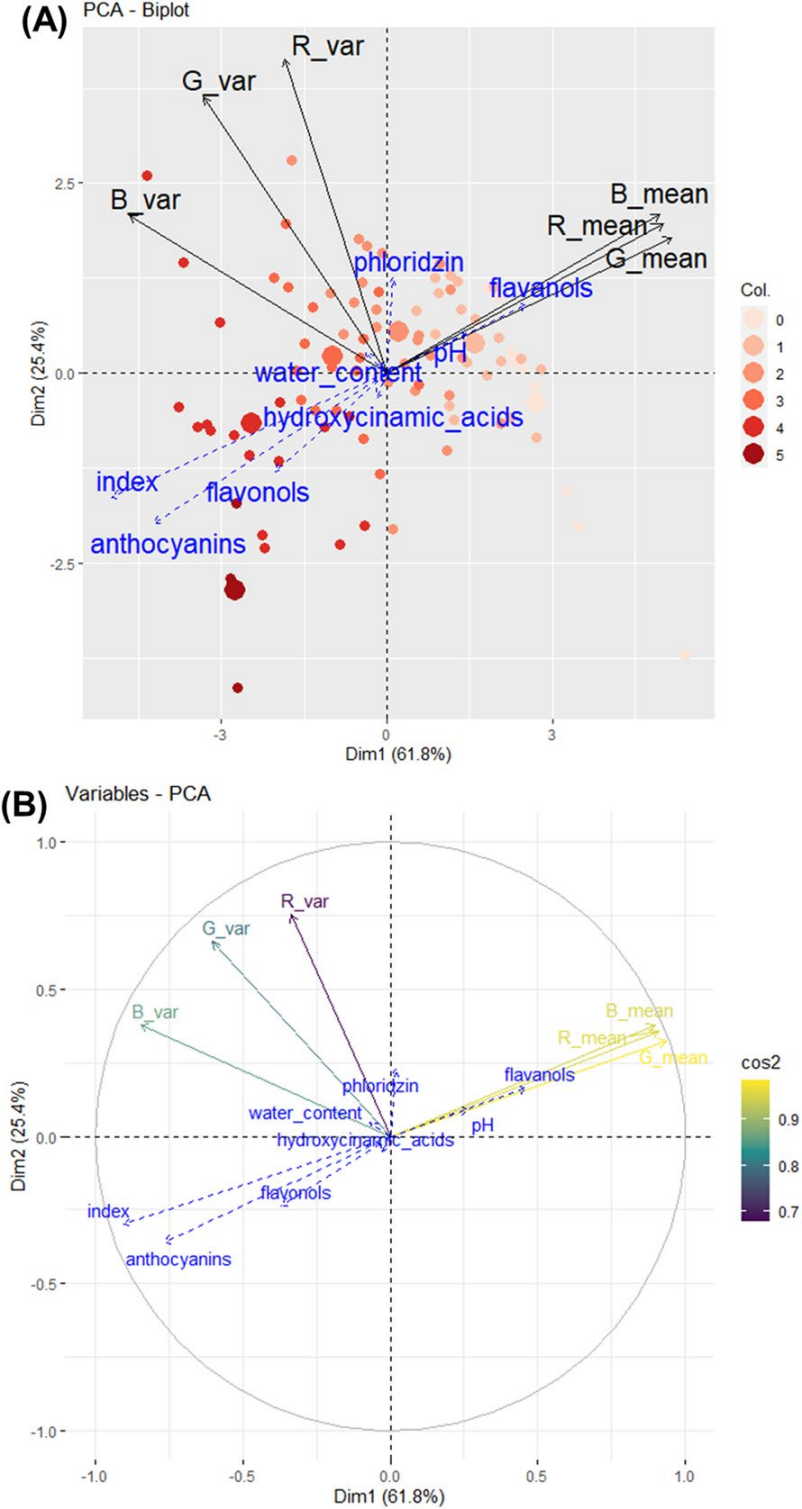
Multivariate analysis based on RGB colour space PCA was performed for colour descriptors of the 91 genotypes. R, G and B mean and standard deviation values were considered as active variables in component calculation. Biochemical factors and colour index values were added as supplementary variables. Data were normalized before performing PCA (feature scaling). **A**: The biplot shows the PCA scores of the explanatory (in black) and supplementary (in blue) variables as vectors. Individuals are coloured according to colour index. Six bigger dots represent barycentres of colour groups. **B**: Correlation circles of colour variables. Arrow colour indicates the cos2 of each explanatory variables on dimensions 1–2

Colour descriptors from RGB and CIEL*a*b* colour spaces were calculated from four fruits per genotype (Table S2). In RGB colour space, red-coloured fruits were associated with low R, G and B values (Fig. 2). The green channel exhibited more variations for mean and standard deviation values. Considering CIEL*a*b* colour space, L* and a* parameters exhibited more variations than b*. Highly-coloured fruits were associated with high a* values and low L* values. Moreover, standard deviation of a* values exhibited more variations than L* and b* channels. Multivariate analyses of colour descriptors from RGB and CIEL*a*b* colour space were performed. Our aim was to confirm the possibility of converting RGB into CIEL*a*b* data and to evaluate the ability of the two colour spaces to distinguish genotypes for the red-flesh pigmentation. A PCA was performed on RGB colour descriptors. First and second axis accounted for 61.8% and 25.4% (Fig. 3) of the total variation, respectively. Axis 1 separates genotypes according to flesh colour (colour index) while axis 2 mostly separates the homogeneous from the heterogeneous flesh colour genotypes. However, less-coloured genotypes (colour index 0,1 and 2) were closely grouped. R, G and B values were strongly correlated to each other and negatively correlated to colour index.

R, G and B colour descriptors showed h^2^ values between 2022 and 2023 image data (Table 2, raw image data: Table S2) with h^2^ values of 0.755,0.783 and 0.763, respectively. For CIEL*a*b* colour descriptors, L*, a* and b* values were 0.786, 0.787 and 0.744, respectively. Visual notation exhibited lower broad-sense heritability than RGB and CIEL*a*b* colour descriptors with h^2^ values of 0.67.

**Fig. 4:**
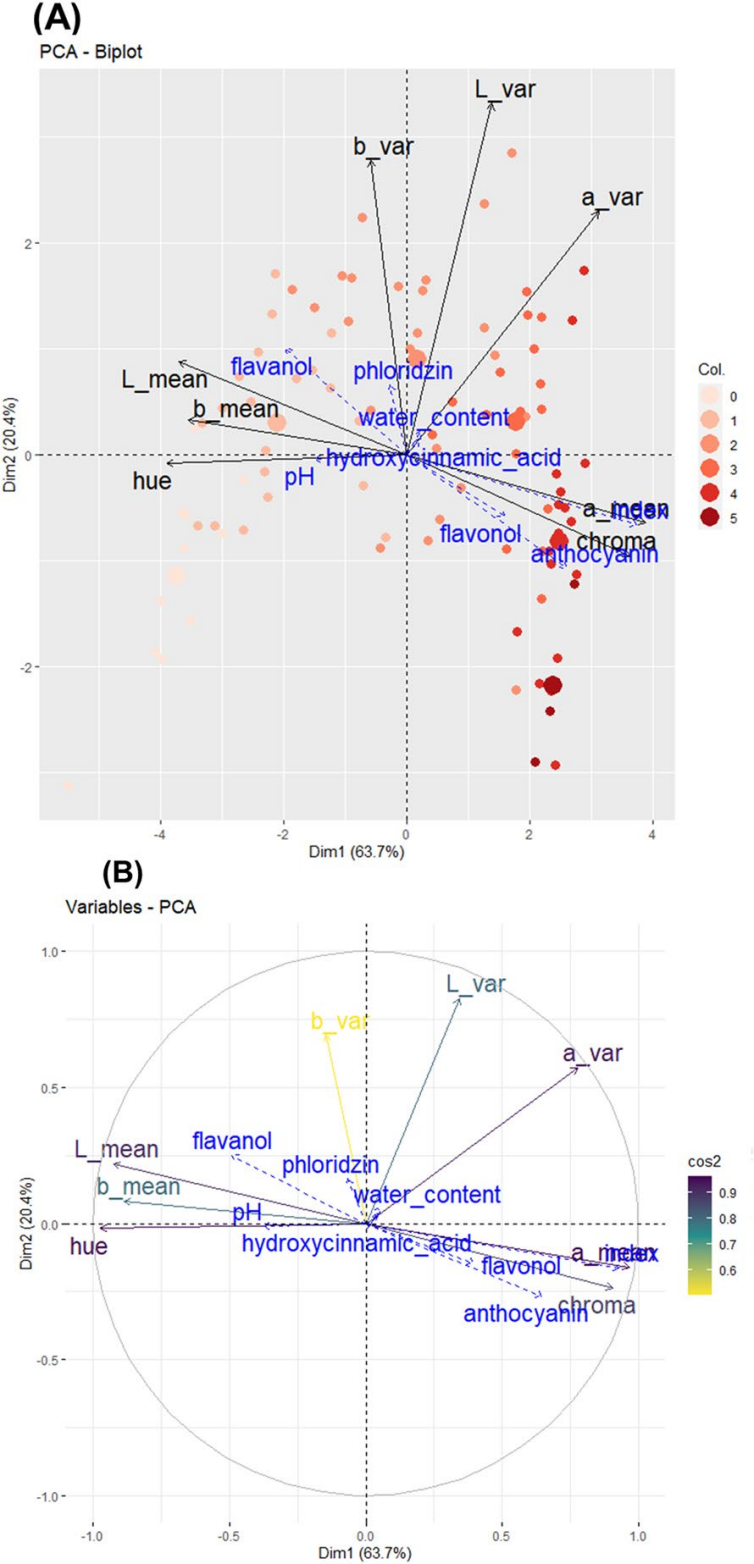
Multivariate analysis of CIEL*a*b* colour space Principal component analysis (PCA) was performed for colour descriptors of the fruits of the 91 genotypes. L*, a* and b* mean and standard deviation were considered as active variables in component calculation. Biochemical factors and index were added as supplementary variables. Data were normalized before performing PCA (mean scaling). Representation of each genotype on the biplot is coloured according to colour index. **A**: The biplot shows the PCA scores of the explanatory (in black) and supplementary (in blue) variables as vectors. Individuals are coloured according to colour index. Six bigger dots represent barycentres of colour groups. **B**: Correlation circles of colour variables. Arrow colour indicates the cos2 of each explanatory variables on dimensions 1–2

**Fig. 5:**
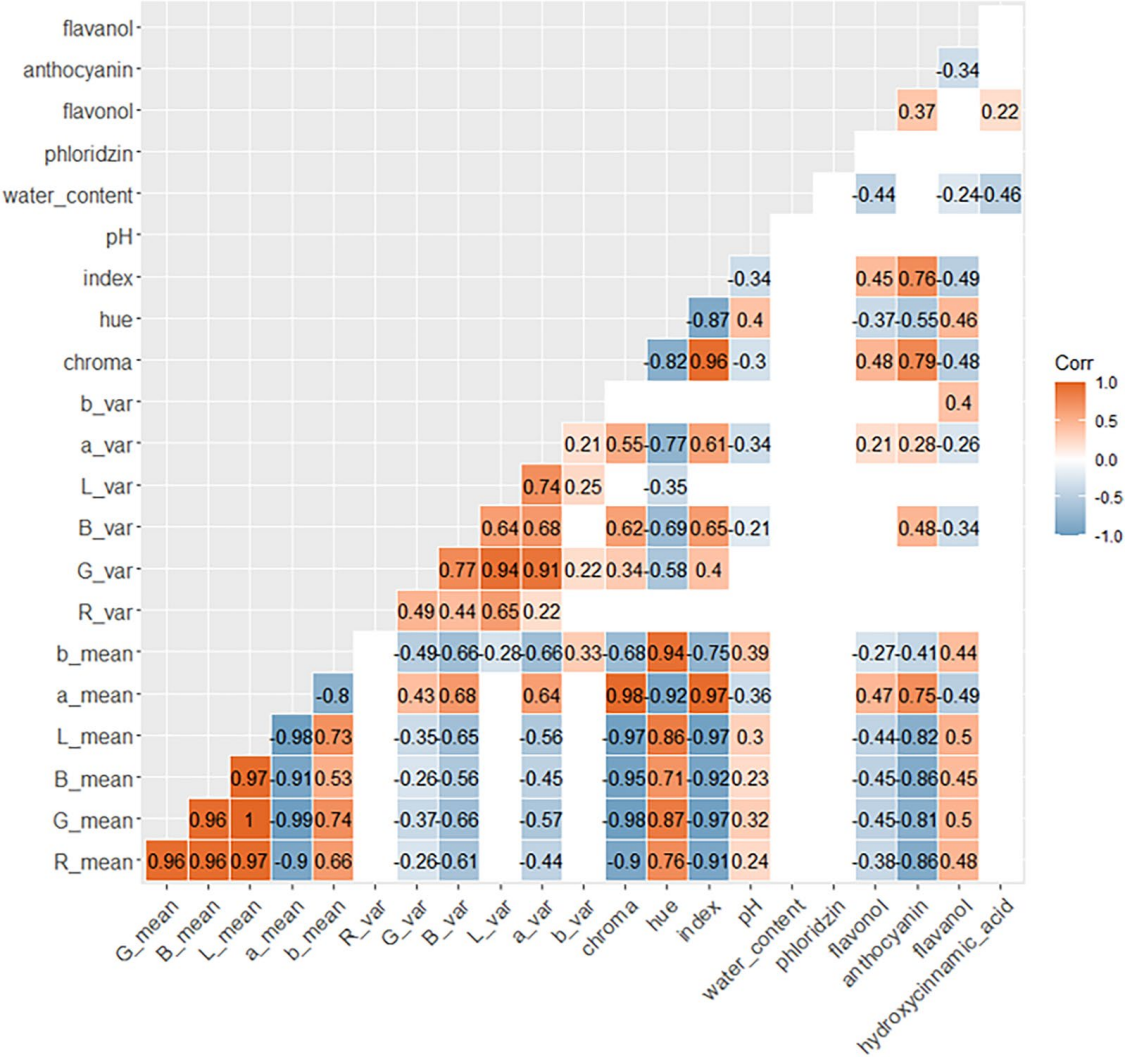
Pearson correlation matrix of biochemical factors and colour descriptors Pearson correlations between biochemical factors and colour descriptors. Only significant values were considered (P $< 0.05$). Positive correlations are highlighted in orange, while negative correlations are highlighted in blue

Considering CIEL*a*b* colour space, red-flesh fruits were associated with high a* and chroma values, and low L*, b* and hue values (Fig. 2). Our results are consistent with CIEL*a*b* colour space definition [36], a* and b* values characterised respectively green to red and blue to yellow variations. Standard deviation values of a* and L* channels exhibited more variations, higher values should be associated with non-homogeneously pigmented phenotypes. Chroma indicated saturation of colour and was correlated to a* and colour index (Pearson correlation of 0.98 and 0.96, respectively - Fig. 5). Principal component analysis based on colour decriptors from the CIEL*a*b* colour space permitted to distinguish genotypes according to their flesh colour index. First and second axis accounted for 63.7% and 20.4% (Fig. 4) of the total variation, respectively. Hue values, ranging from red (0) to yellow (60), exhibited a negative correlation with axis 1. Concomitantly, within the RGB colour space, axis 1 effectively distinguished genotypes based on red-flesh colour (colour index), while axis 2 discriminated between genotypes characterized by homogeneous and heterogeneous pigmentation.

### Phenolic compound composition and biochemical factors

Several biochemical parameters (phenolic compound contents, dry matter content and pH values) were measured to evaluate their influence on colour expression (Fig. 5A). Polyphenol concentrations are expressed in $\upmu$g.g^-1^ of fresh weight (FW) and summarised in total content per class (Table S1, Fig. 5B, C). Relative standard deviation varied from 0.78 to 4.89 with an overall mean of 2.74 when considering the two parents for the ten phenolic compounds (Table S3). Accordingly, two replicates for each genotype (two distinct metabolites extractions) were analyzed and the metabolite content was expressed as the mean of these two values. Hydroxycinnamic acids, among all measured compounds, reached the highest concentrations: from 7.92 to 141.07 and from 49.93 to 804.25 $\upmu$g.g^-1^ of FW for 4-*p*-coumaroylquinic acid and chlorogenic acid, respectively. Anthocyanins were represented by cyanidin 3-galactoside and, as expected, their content varied greatly among our samples: from 2.37 to 309.47 $\upmu$g.g^-1^ of FW. It was followed by flavanols: between 0.45 and 64.59, 2.02–194.16, 0.21–49.47, 2.96–282.59, 2.32–123.79 $\upmu$g.g^-1^ of FW for (+)-catechin, (−)-epicatechin, procyanidin B1, procyanidin B2, and procyanidin C1, respectively. Phlorizin (class of dihydrochalcone) contents ranged from 0.63 to 32.38 $\upmu$g.g^-1^ of FW. Finally, within the flavonol group, quercetin 3-galactoside was the most concentrated: contents ranged from 1.26 to 11.65 $\upmu$g.g^-1^ of FW. Water contents and pH varied among individuals with a mean water content of 81.5% (range from 76.5% to 91.0%) and a mean measured pH value of 3.18 (range from 2.91 to 3.83). Red-flesh colour showed a clear relationship to anthocyanin contents (Pearson correlation = 0.75) and confirmed the possibility to estimate anthocyanin contents from red colour [55]. However, logarithmic evolution of anthocyanin contents suggests the existence of a saturation point. Beyond this, the red colour intensity does not change and could lead to underestimation of anthocyanin contents in a linear model.

### Correlation between biochemical factors and colour data

Pearson correlations were calculated to study association between biochemical factors and colour descriptors. As observed in RGB-PCA (Fig. 3), R, G and B channels were highly correlated (correlation = 0.96). RGB descriptors were also negatively correlated to colour index (from $-$0.91 to $-$0.97) and anthocyanin contents (from $-$0.81 to $-$0.86).

L*a*b* and RGB colour descriptors were highly correlated with absolute values oscillating between 0.91 and 1 for L* and a* parameters. b* exhibited lower values with correlations of 0.66, 0.75 and 0.53 for R, G and B parameters respectively. a* parameter was strongly correlated to colour index (0.97) and anthocyanin contents (0.75).

**Fig. 6:**
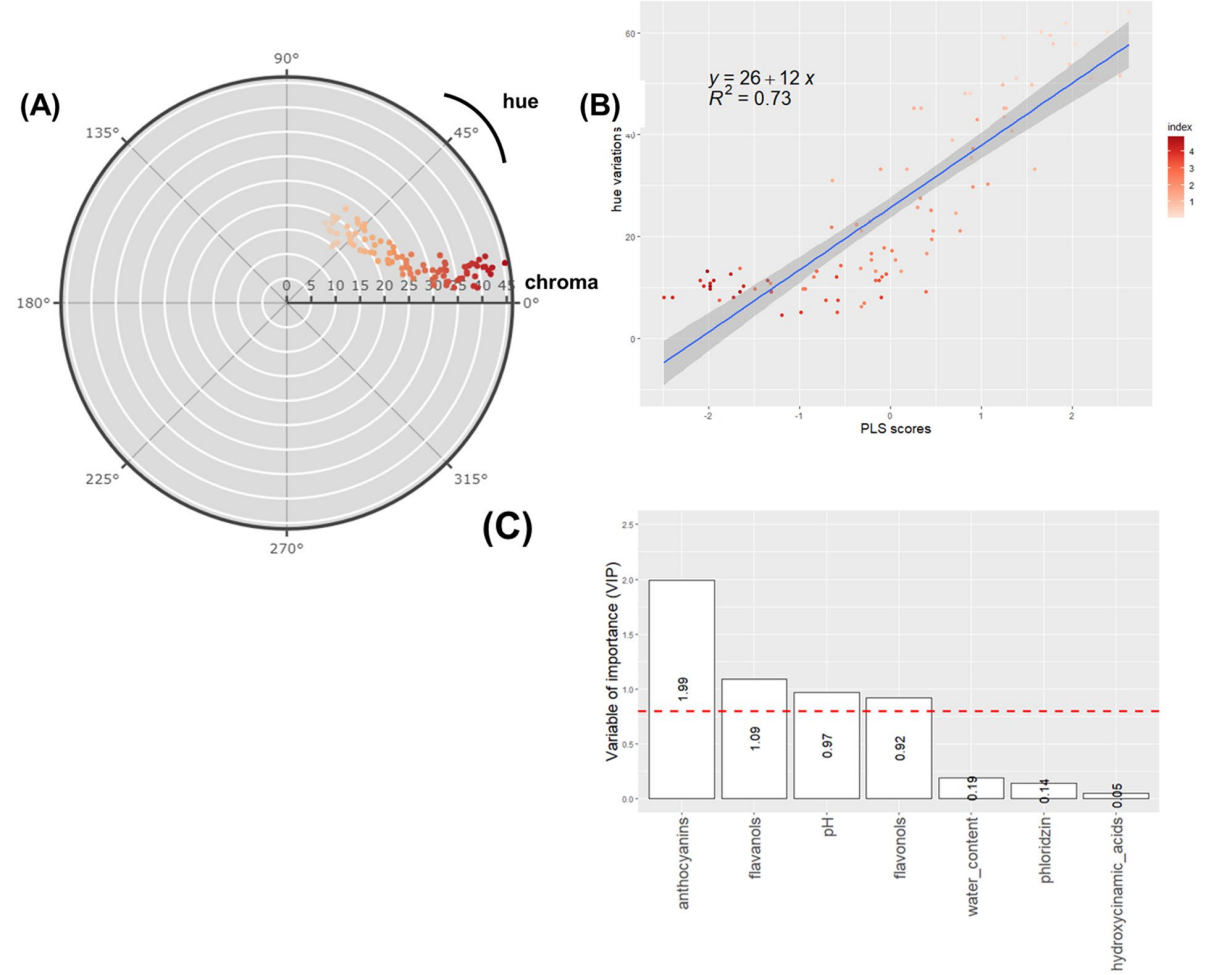
Modelling of hue colour by biochemical factors Supervised multivariate analyses were performed using Partial Least Square (PLS) regression. Hue was considered as a response variable (Y) and biochemical factors as predictors (X). **A**: Circular plot showing distribution of hue and chroma. Dots are coloured according to hue. **B**: Scatter plot of observed hue and predicted values of the PLS model. Dots are coloured according to their hue from green (lower values) to red (higher values). **C**: Variable of importance (VIP) scores. **A** variable with a VIP value above 0.8 is a variable that appears to have a considerable impact on the model

### Modelling of colour descriptors with biochemical factors

**Fig. 7:**
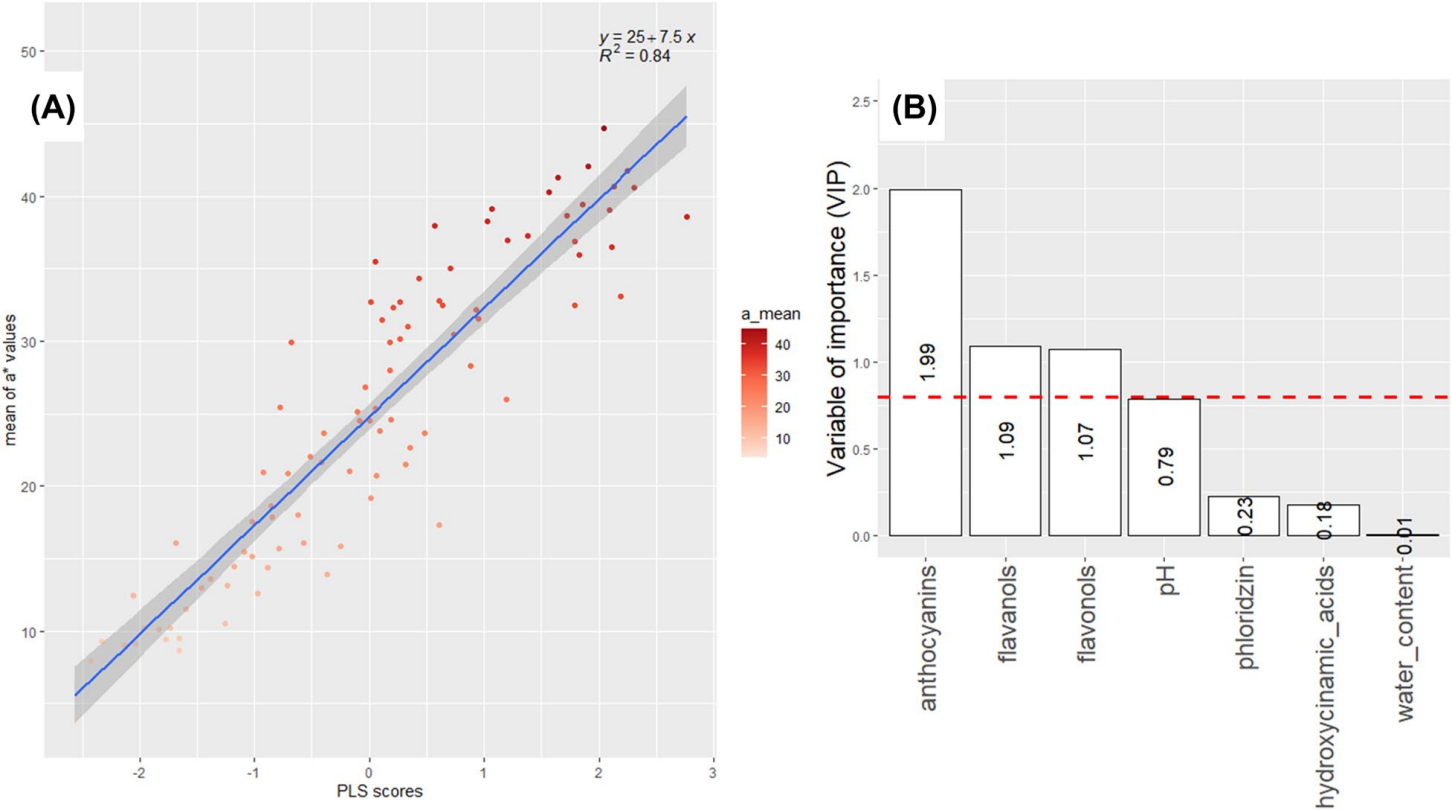
Modelling of a* by biochemical factors Supervised multivariate analyses were performed using Partial Least Square (PLS) regression. a* values were considered as response variable (Y) and biochemical factors as predictors (X). **A**: Scatter plot of observed a* and predicted values of the PLS model. Dots are coloured according to a* from green (lower values) to red (higher values). **B**: Variable of importance (VIP) scores. **A** variable with a VIP value above 0.8 is a variable that appears to have a considerable impact in the model

**Fig. 8:**
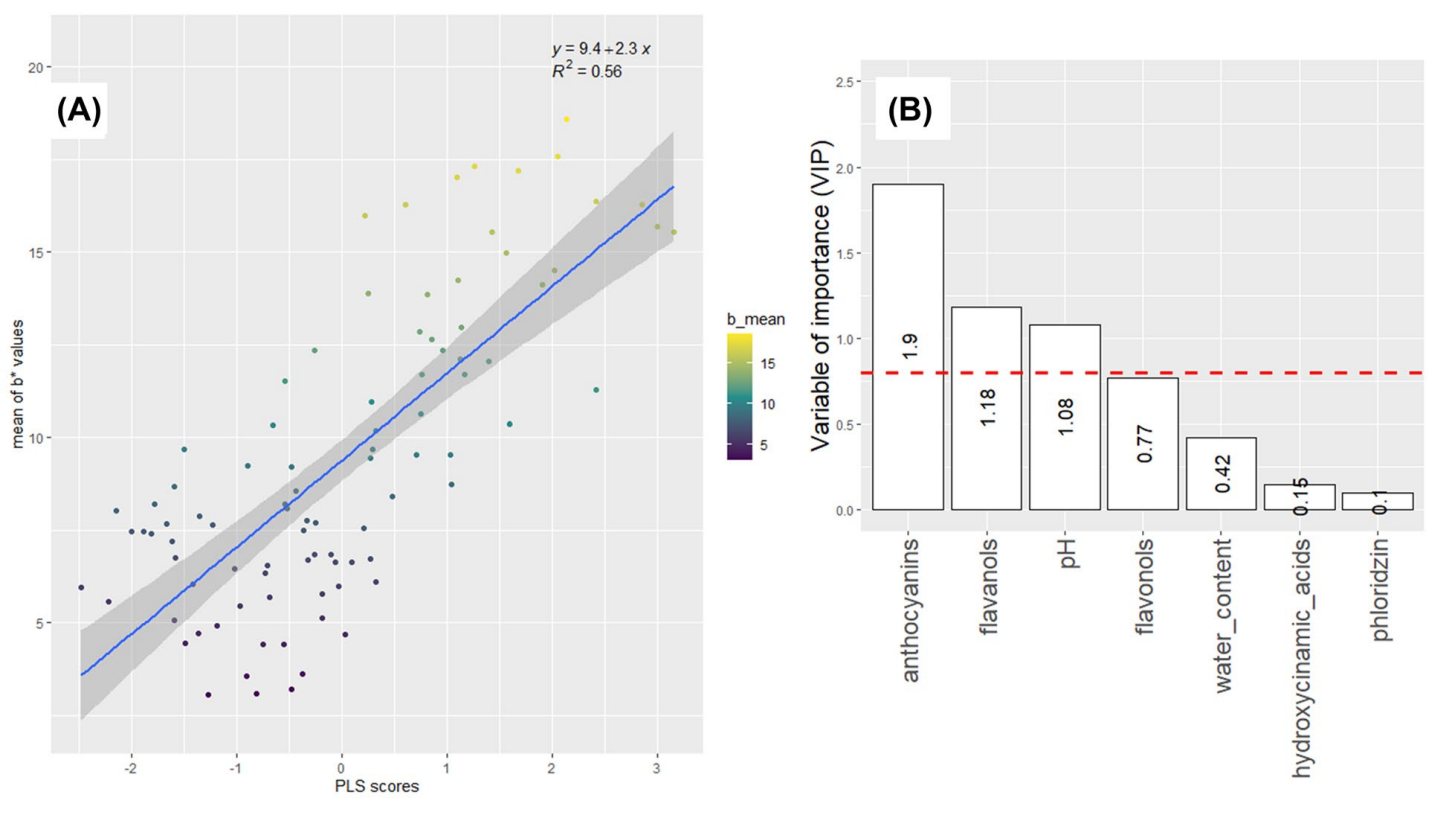
Modelling of b* by biochemical factors Supervised multivariate analyses were performed using Partial Least Square (PLS) regression. b* values were considered as response variables (Y) and biochemical factors as predictors (X). **A**: Scatter plot of observed b* and predicted values of the PLS model. Dots are coloured according to b* from blue (lower values) to yellow (higher values). **B**: Variable of importance (VIP) scores. **A** variable with a VIP value above 0.8 is a variable that appears to have a considerable impact on the model

Hue values (Fig. 6A) distribution was coherent with colour variations from white-off to red [72]. Hue was expressed through a single component model (*R*2 = 0.73 and RMSE = 6.92 - Fig. 6B) which combine predictor variables found in a* and b*: Anthocyanin, flavanol, flavonol contents and pH have VIP values of 2.02, 1.08, 0.88 and 1.97, respectively (Fig. 6C). One component was determined for a* values with *R*2 = 0.84 (Fig. 7A) and RMSE = 3.26. Anthocyanin, flavanol and flavonol contents had the greatest impact on the model construction with VIP values equal to 2, 1.1 and 1.04, respectively (Fig. 7B). One component was also sufficient for b* values to capture most of the inertia with *R*2 = 0.56 (Fig. 8A) and RMSE = 2.27. Anthocyanin, flavanol contents and pH were significant in the model construction with VIP values equal to 1.92, 1.18 and 1.07, respectively (Fig. 8B).

## Discussion

### Distribution of the contents of phenolic compounds

Total phenolic compounds and individual concentrations (Table S1) were consistent with those observed in some wild apple species [73] or cider apples [74, 75]. We have to consider that the genetic background of red-fleshed apple varieties is close to that of the wild apple species [15].

As expected, anthocyanin contents varied greatly among our samples. Some genotypes displayed higher anthocyanin concentrations, with anthocyanin contents superior to 100 $\upmu$g.g−1 of FW for 19 samples, than those found in literature [21, 22, 63]. Indeed for these particular genotypes, anthocyanin content was comparable to that of other anthocyanin-rich species such as strawberry (*Fragaria × ananassa*) or blueberry (*Vaccinium sp.*) [76]. These results suggest that red-flesh apples are an interesting natural food source of anthocyanins. However, these contents could have been overestimated (in comparison to whole fruit) by our sampling method: we always selected flesh tissues from the sun-exposed side of the fruit to allow comparison between genotypes. The sun-exposed side of apple fruits is generally more concentrated in phenolic compounds [22].

### Dissection of the red-flesh trait in the CIEL*a*b* colour space

In this study, we confirmed the transferability of RGB to CIEL*a*b* coulour space and the suitability of these two colour spaces with visual notation. Both colour spaces were well-adapted in discriminating genotypes. Moreover, colour descriptors were more accurate than visual notations because of their higher broad-sense heritability values. Indeed, colour index broad-sense heritability was 0.67 while values were comprised between 0.744 and 0.787 for colour descriptors. a* exhibited slightly higher broad-sense heritability than the other colour descriptors.

R, G and B parameters were individually correlated to colour index. Strong associations of RGB variables in PCA space confirm the difficulty of studying and interpreting each of them. This behaviour is relative to additive colour theory. By definition, low R, G or B values taken separately are associated with dark colours. In our study, dark colours corresponded to red-flesh colours. In other anthocyanin-rich species [1], phenotyping colours can be more challenging using RGB colour space. Only considering dark colours could lead to overlap divergence between dark-red and deeper purplish phenotypes. Consequently, considering R, G and B individually will lead to bias in distinguishing these phenotypes, given that, colour in RGB space is defined by mixing R, G and B values together [77].

Interestingly, colour heterogeneity was also repeatable between 2022 and 2023 with h^2^ values comprised between 0.372 and 0.649, indicating a potential genetic control. However, we did not identify any statistical association between colour heterogeneity (L*, a* and b* variance) and particular phenolic contents (data not shown). This lack of association could be attributed to the limited-area in the sampling method which does not distinguish red and white parts in heterogeneous pigmented fruits, or, alternatively, to the regulation of pigmentation heterogeneity by an activator/repressor system during fruit development [78]. Underhill et al. [38], argued that a major challenge in phenotyping grape berry colour is the classification of fruits into discrete classes despite a continuous variation within and among genotypes. Our image analysis pipeline enables relative colour comparison by considering each apple image as pixel matrices and then estimates statistical descriptors. The use of quantitative colour measurements rather than qualitative categorisation could help in detecting minor QTL related to flesh colour variations [39].

### Identification of biochemical factors involved in red pigmentation

PLS regression models enabled identification of important biochemical factors involved in colour expression. Altogether, we found that anthocyanins, flavonols, flavanols and pH were involved in hue variations and, therefore, in red-flesh colour expression with model coefficients of $-$0.51, $-$0.22, 0.27 and 0.24, respectively. a* colour variation was associated with anthocyanins, flavonols and flavanols (Fig. 7). The importance of non-anthocyanin compounds in red colour expression supported copigmentation events where flavonols could be copigments of anthocyanins, leading to a hyperchromic shift providing a deep red colouration [26]. In contrast to anthocyanins and flavonols, flavanols were negatively associated with higher a* values (coefficients of 0.53, 0.28 and $-$0.29, respectively). Distribution of flavanol and anthocyanin contents suggested an imbalance between anthocyanin and flavanol accumulation. Indeed, genotypes that exhibit redder fruits tend to have lower (8-fold) flavanol contents than non-red fruits, which would be consistent with a competition between two end-products of the flavonoid biosynthetic pathway [79]. Other studies on fruit berries, reported that increased anthocyanin accumulation results in a lower non-anthocyanin phenolic production because of the competition for the same substrates [80, 81]. Moreover, anthocyanin degradation occurrs more rapidly in the presence of flavanols and could decrease the chemical stability of flavonol/anthocyanin solutions [82]. Knowing the prevalence of Flesh Browning Disorder (FBD) in red-flesh cultivars [83], decorrelation of flavanol and anthocyanin production could help in breeding red-flesh cultivars less prone to develop FBD.

Interestingly, b* variation was linked to anthocyanins, flavanols and pH with model coefficients of $-$0.42, 0.26 and 0.24, respectively, highlighting the importance of vacuolar pH in colour expression [84, 85]. The lowest pH values were associated with the lowest b* values and therefore to a blue colour. At similar anthocyanin levels, a more acidic vacuolar environment could lead to a chromatic shift from red to purple, resulting in a deeper colouration. During fruit development, anthocyanin contents in the flesh decrease [86]. This phenomenon is well known as the dilution process and could lead to a decrease in colour intensity. However, water content does not seem to be involved in red-flesh colour differences among mature fruits when compared at the same developmental stage.

### Applications of colour analysis in red-flesh breeding

One of the main challenge in plant breeding is the development of robust phenotyping methods to accurately measure large number of plants in an increasingly constrained environment (e.g. time, cost and data management) [87]. Fruits are plant organs that are prone to damages and biochemical degradation, making fast and portable acquisition systems a necessity to reduce time from harvest to feature acquisition. Our acquisition system provides fast image acquisition (10 s/image) under repeatable lightning conditions (LED). Moreover, this system requires low storage capacity (200 Ko/image, 569 Mo for 3402 images) facilitating a deployment close to field trials. It provides a robust and accurate method for evaluating flesh colour and may be deployed for other species.

The rapid advancements in spectroscopy [88], coupled with the identification of key biochemical factors influencing red-flesh colour in apples, offer the potential to develop models capable of predicting phenolic compound contents. This would circumvent the need for labor-intensive, expensive, and time-consuming laboratory-based steps. However, characterisation of non-homogeneous surface remains a challenge in spectroscopy due to the limited acquisition area. The integration of hyperspectral imaging [89] and RGB-colour analysis holds promise for assessing the phenolic profile at a pixel scale. Extending this methodology to other F1 families of red-flesh apples will help to better understand the genetic determinism of phenolic profiles and colour expression in other red-flesh pedigree.

## Conclusion

In this study, we designed a Python pipeline to extract colorimetric descriptors from RGB images acquired with a flatbed scanner. This pipeline can be easily implemented in more versatile image analysis tools like “PhenoBox” [90]. We confirmed RGB and CIEL*a*b* colour spaces efficiency in discriminating genotypes in comparison with visual notation. Acquisition of biochemical data associated with colour analysis enabled the identification of relevant biochemical factors involved in flesh colour in apple. These analyses revealed that anthocyanin, flavonol and flavanol concentrations, as well as pH are closely related to hue variations, highlighting the multifactorial determinism of the red-flesh trait. Altogether, these results will help in deciphering the genetic determinism of red-flesh trait in apple.

### Supplementary Information

Supplementary file 1.

## Data Availability

The datasets generated and/or analysed during the current study are available in supplementary files (Table S1 and S2). Script designed for image analysis is public and can be found at: https://github.com/pibouillon/colour_val/blob/main/colour_val.py
